# Anti-pathogenicity of *Acanthus ilicifolius* leaf extracts against *A. hydrophila* infection in *Labeo rohita* fingerlings

**DOI:** 10.1186/s13568-023-01595-y

**Published:** 2023-08-20

**Authors:** M. V. N. Sravya, G. Simhachalam, N. S. Sampath Kumar, K. Govindarao, T. Rahul Sandeep, D. Divya

**Affiliations:** 1 Department of Zoology and Aquaculture, Acharya Nagarjuna University, Guntur, Andhra Pradesh 522510 India; 2 Department of Biotechnology, Vignan’s Foundation for Science, Technology and Research, Vadlamudi, Andhra Pradesh 522213 India

**Keywords:** *Acanthus ilicifolius*, Antibacterial activity, Antioxidant activity, GC–MS, *L. rohita*

## Abstract

Antibiotic resistance has become one of the inevitable barrier in aquaculture disease management. Herbal drugs has evolved to be the novel ways of combating drug resistant pathogens. In the current investigation, leaf extracts of mangrove plant, *Acanthus ilicifolius* were assessed for in vitro studies, among the selected four extracts, methanol extract has expressed highest antibacterial activity against *P .aeruginosa* (4 ± 0.3 mm), *A. hydrophila* (5.9 ± 0.5 mm), *S. aureus* (3.5 ± 0.7 mm) and *B. subtilis* (2.9 ± 0.5 mm) and antioxidant activity, DPPH (81.3 ± 1.0 AAEµg/ml) and FRAP (139.1 ± 1.5 AAEµg/ml).TPC and TFC were higher in the methanolic extract and has exhibited positive correlation with both DPPH and FRAP assays. Considering the in vitro efficiency, methanol extract was purified successively by column and thin layer chromatography and characterisation by GC–MS unveiled the presence of 2-Propanethiol, Trimethylphosphine, Pentanoyl chloride, Dimethylhydroxymethylphosphine and Propanedinitrile, ethylidene. *A. hydrophila* infected *L. rohita* fingerlings has survival percentage 81% and 94% in extract treated groups over 0% in negative control and 71% in positive control.

**Supplementary Information:**

The online version contains supplementary material available at 10.1186/s13568-023-01595-y.

## Introduction

Over the past two decades aquaculture has received huge attention as one of the rapidly developing food producing industries (Edwards et al. 2019). Economically it has been supporting 10–12% of global population (FAO 2014). Freshwater aquaculture accounts 95% of the total aquaculture production in India (DADF 2017), of which the carp culture is most preferred due to its fast growth and consumptive value (Ngasotter et al. 2020). Fin fish and shell fish has become invaluable sources of protein and essential nutrients in the human diet of day to day life (Garlock et al. 2020). In the recent past, the demand for the fish protein was increased massively. In order to meet the demand, the culture practices were progressed towards semi intensive and intensive methods. Aquaculturists are ensuing vertical expansion of the culture with high stocking densities which leads to water quality deterioration, stress, immune suppression in the host and spread of infectious diseases (Mishra et al. 2017; Dawood 2021). Antibiotics of class beta lactams, tetracyclines, quinolones, sulphonamides, aminoglycosides, lincosamides, nitrofurans, chloramphenicols and macrolides (Sun et al. 2020) are mainly destined to treat the bacterial infections (Miranda 2012). Frequent use of these antibiotics as therapy, metaphylaxis and prophylaxis will eventually results in the development and propagation of multidrug resistant genes in the bacteria, effects non-target organisms, accumulation of residues in the fish tissues and surrounding environment (Okoye et al. 2022). The drug resistant genes and antibiotic residues get disseminated to the consumer (Heuer et al. 2009; Done et al. 2015) and impose perturbation of intestinal microbiota, mutagenicity, hypersensitivity, aplastic anaemia and carcinogenicity (Liu et al. 2017).

Phytocompounds has become a promising therapeutic alternative in the place of antibiotics (Zhang et al. 2022), they are biodegradable, eco-friendly, treats the disease without instigating anti-drug resistance (Direkbusarakom 2004). The phytocompounds enhances digestion, feed conversion ratio, enzyme activity, protein synthesis, phagocytic activity, healing power, immunity (specific and non-specific) in the fish. Mangroves are obligate halophytic, stress tolerant plants (Wang et al. 2011) with structurally diverse and unique bioactive compounds, act as prospective source for the development of novel therapeutic products (Alvin et al. 2014).

Phytocompounds of *Acanthus* species possess remarkable pharmacological properties viz*.,* anti-microbial, anti-inflammatory, antioxidant, antinociceptive, anti-parasitic. *Acanthus ilicifolius* L. vernacularly known as Alasyakampa, Alchi of order Magnoliopsida and family Acanthaceae with spiny lanceolate leaves and can grow up to a height of 3 m. Traditionally, the plant has been used for dyspepsia, paralysis, asthma, headache, rheumatism, and skin diseases (Patel et al. 2020; Matos et al. 2022). The current work focused on in vitro and in vivo application of bioactive compounds of *A. ilicifolius* against *Aeromonas hydrophila* and also studied free radical scavenging efficiency.

## Material and Methods

### Plant collection and extraction

The present study was aimed to assess the efficacy of phytocompounds in *A.ilicifolius* leaves as a therapy for bacterial diseases in fish culture. Fresh leaves were collected on 12.04.2021 from Gilakaladindi mangrove forest (16.14° N, 81.16° E), 6 km East to Machilipatnam, Andhra Pradesh, India. *A*.*ilicifolius* confirmation was done by taxonomist from the Dept. of Botany and documented (AI-142021) in the museum, Acharya Nagarjuna University, Andhra Pradesh, India. The remnants of dust particles were removed by washing and leaves were shade dried. The desiccated leaves were ground to fine powder, mixed with petroleum ether, ethyl acetate, methanol and double distilled water in a ratio of 1:10 (w/v), extracts were prepared by soxhlet technique maintained at suitable temperature for 7 h. Filtered extracts (Whatman No.1) were concentrated by using rotary evaporator.

### Qualitative analysis of phytochemicals

Standard protocols of (Harborne 1998) were followed to detect the presence of phytochemicals (saponins, tannins, flavonoids, steroids, terpenoids, glycosides, carbohydrates, proteins, phytosterols, cardiac glycosides, alkaloids, phenols, reducing sugars, anthraquinones) in leaf extracts.

### Agar well diffusion assay and minimum inhibitory concentration

The method of (Rios et al.^1988^) was adopted for determining the antibacterial activity of the leaf extracts. 100 µl each of *Pseudomonas aeruginosa* (MTCC1934), *Aeromonas hydrophila* (MTCC1739), *Staphylococcus aureus* (MTCC1430) and *Bacillus subtilis* (MTCC121) were inoculated individually on solidified Mueller Hinton Agar (MHA) petri plates. The wells (6 mm diameter) were bored by sterile cork- borer and filled with 40 µl of leaf extracts and oxytetracycline of 20 µg/ml concentration which was used as positive control. The inhibitory zones (mm) were measured after 24 h incubation at 37 °C.

(Benger et al. 2004) protocol of broth dilution was adopted for the minimum inhibitory concentration (MIC). To the sterile tubes with 10 ml of MHA broth, the methanol leaf extract of *A.ilicifolius* leaf was added in a serial concentration of 10 to 100 µg/ml except for the control. 0.5 ml (1 × 10^6^ CFU) *A.hydrophila* was added to each tube. After 24 h of incubation at 37 °C, the concentration of the extract that inhibited visible bacterial growth was noted as the MIC.

## Antioxidant activity

### Total phenol content (TPC) and total flavonoid content (TFC)

For TPC, Folin-Ciocalteu protocol (Ali et al. 2014) was followed. 1 ml of 0.5N folin-ciocalteu reagent was added to 250 µl of the sample and left undisturbed for 5 min. To this 1.5 ml of 20% (w/v) sodium carbonate solution was added, vortexed and incubated for 90 min at 37 °C. The absorbance was noted at 760 nm. TPC of the leaf extract was presented as mg GAE/g DW (mg gallic acid equivalent per gram dry weight).

TFC was determined by aluminum chloride colorimetric method (Koolen et al. 2013). 250 µl of the extract was mixed with 2% methonolic aluminum chloride (250 µl), incubated for 45 min at room temperature and the absorbance was measured at 430 nm in UV–vis spectrophotometer. TFC was expressed as mg rutin equivalent per gm of body weight (mg RE/g DW).

### DPPH radical scavenging assay

(Blios 1958) method of DPPH assay was followed for the determination of antioxidant activity. Known quantity of DPPH solution was mixed with 31.25, 62.5, 125, 250, 500 µg/ml of leaf extracts, incubated in dark for 30 min. At 517 nm, absorbance of the coloured solution was measured. Methanol and ascorbic acid were used as the blank and standard respectively. The percent DPPH radical scavenging activity of the leaf extracts was calculated using (A0-A1)/A0 × 100, A0 and A1 are the absorbance of the control and sample respectively.

IC_50_ of leaf extracts against DPPH free radical was obtained by plotting a graph between concentration and % free radical scavenging activity of the extract, the value was calculated by interpolation of linear regression analysis.

### FRAP assay

(Benzie and Strain 1996) protocol was followed for the FRAP assay. The FRAP reagent, 1 volume of 20 mM FeCl_3_ and 1 volume of 40 mM HCl containing 10 mM 2,4,6-tri (2-pyridyl-s-triazine) (TPTZ) were added to 10 volume of 300 mM of acetate buffer (1:1:10 v/v). To this reagent 15.62, 31.25, 62.5, 125, 250, 500 µg/ml of leaf extracts were added, incubated for 15 min at 37 °C. At 593 nm the optical densities were measured. Ascorbic acid was standard and the results for both DPPH and FRAP were noted as µg of AAE per ml.

## Separation and purification of the extracts

### Column chromatography

Methanol leaf extract (20 g) of *A*.*ilicifolius* were passed through the column packed with 100 to 200 mesh sized silica gel. The mobile phase acetone:methanol (9:1 to 1:9) were used for the elution of the sample. Fractionation of the samples was carried by gradient separation of the mobile phase. The similar coloured elutes were pooled, labelled and evaluated for the antibacterial and antioxidant activity.

### Preparative TLC

The active fractions that yielded high antibacterial and antioxidant activity were further separated and purified by preparative TLC. The glass plates (250µ thickness, 20 × 20 cm) were coated with slurry; 30 g of silica gel and small quantity of CaSO_4_ in 60 ml triple distilled water. The plates were dried at room temperature for 10 min, at 105 °C in hot air oven for 1 h and for 2 h in a desiccator.

The known amount of active fraction was placed on TLC plate and was kept in solvent system, 12:6:3 of butanol: acetone: water. The bands formed by the capillary movement of the solvent were observed under UV light illumination. The procedure was repeated to get the bands with good resolution and in multiple number. The multiple scrapings of similar Rf value bands were pooled and tested for both antibacterial and antioxidant activity.

### GC–MS

GC–MS analysis was carried out using an Agilent (5977B) single quadrupole GC/MS followed towards mass detector. Absorbed silica capillary column made of 95% dimethylpolysiloxane and 5% phenyl (HP-5MSI; length: 90 m, diameter: 0.250 mm and foil: 0.25 m). Maintained injector temperature of 25 °C, a fontal oven temperature of 90 °C, a gradual increase to 200 °C at a rate of 3 °C/min for 2 min, and a final increase to 280 °C at a rate of 15 °C/min for 2 min. Helium 99.999% was the carrier gas with a column flow rate of 1 µm /min. The fixed interface temperature between GC and MS was set to 280 °C, and the electron ionisation system on the MS was in scan mode. The examined mass range was between 50 and 550 m/z, and the MS quad and source temperatures were computed at 250 °C and 280 °C, respectively. NIST spectral library was utilized to look for and identify each component.

## In vivo studies

### Experimental design

Pathogen free healthy *Labeo rohita* fingerlings, 9.5 ± 0.5 cm and 11 ± 0.9 g were separated from the random sample, acclimatised in 40L capacity tubs with 24 h aeration for 10 days prior to the experimentation. Fishes were fed twice daily with commercial feed of 5% bodyweight. From the bottom of tubs, leftover feed was siphoned out and 1/3^rd^ of the water was replaced at late afternoon to reduce water toxicity caused by excreta (ammonia).

On the 11th day, acclimatised healthy fingerlings were segregated into five groups with nine in each (n = 3). Except control all the experimental groups were starved for 24 h.

Group I: Control (Neither infected nor treated)

Group II: Negative control (Infected but not treated).

Group III: Positive control (Infected and treated with Oxytetracycline).

Group IV: Infected and treated with crude leaf extracts.

Group V: Infected and treated with purified leaf extracts.

12^th^ day of the experiment, group II to V infected with 0.5 ml of *A. hydrophila* (10^3^ CFU). Subsequently, from 13^th^ day Group III, IV and V were treated with oxytetracycline 2.5 mg/ kg body weight, crude extract 4 mg/kg body weight and purified extract 2.5 mg/kg body weight of *A*.*ilicifolius* respectively for 6 days. Pathogen and treatment for the fingerlings was administered through feed and dosages were determined by pilot studies.

### Confirmatory tests of *A. hydrophila*

Followed Koch’s postulates criteria; bacteria was isolated from moribund fingerlings, cultured, diseased healthy fingerlings by inoculating the cultured bacteria. Further the bacteria was re-isolated, compared the bacteria used in the present experiment and confirmed *A. hydrophila* as pathogenic. Morphological and biochemical confirmatory tests were conducted for *A. hydrophila* (Gilardi 1967).

### Estimation of catalase (CAT) and superoxide dismutase (SOD)

Hepatic tissue of all the experimental groups were homogenised using 15 mM Tis-HCl buffer at pH 7.4, centrifuged for 20 min at 10,000 rpm, 4 °C and supernatant was used for estimation of protein, CAT and SOD by (Bradford 1976; Aebi 1984; Misra and Fridovich 1972) methods respectively.

0.05 ml of sample was mixed with 1.95 ml phosphate buffer and 1 ml hydrogen peroxide and the decreased absorbance was measured at 240 nm against blank. In the presence of CAT, H_2_O_2_ break down into H_2_O and O_2._ CAT levels were estimated by measuring the decrease in hydrogen peroxide (H_2_O_2_) concentration in the mixture. For estimation of SOD, sample (0.01 µl) was mixed with carbonate buffer (2.96 µl), epinephrine (0.01 µl), EDTA (0.02 µl), incubated for 2 min at room temperature and measured the absorbance at 450 nm. CAT and SOD levels were expressed in units of enzyme per mg of protein.

### Statistical analysis

The relationship between TPC, TFC and antioxidant activity (FRAP and DPPH) were calculated by Pearson’s correlation coefficient. SPSS software was used for the statistical analysis.

## Results

### Qualitative analysis of Phytochemicals

*A.ilicifolius* leaf extracts disported positive results for all the tested phytochemicals except for anthraquinones (Table 1). Maximum number of phytochemicals got extracted into the methanol.

**Table 1:** Qualitative analysis of phytochemicals in *A.ilicifolius* leaf extracts in solvents

Phytochemicals	Petroleum ether	Ethyl acetate	Methanol	Aqueous
Saponin	−	+	+	+
Tannin	−	-	+	-
Flavonoid	+	+	+	+
Steroid	+	−	−	−
Terpenoid	+	−	−	−
Glycosides	−	+	−	+
Carbohydrates	−	−	+	−
Proteins	+	−	+	−
Phytosterols	+	−	+	+
Cardiac glycosides	+	+	+	+
Alkaloid	+	-	+	+
Phenols	+	+	+	+
Reducing sugars	−	−	+	+
Anthraquinones	−	−	−	−

+ presence, −absence

The results of the work were presented in mean ± S.D, with n = 6 .

### Antibacterial activity and minimum inhibitory concentration

The antibacterial activity of the extracts exhibited varied inhibitory zones against gram positive (*S. aureus* and *B.subtilis*) and gram negative bacteria (*P.aeruginosa* and *A. hydrophila*) (Additional file 1: Table S1). Highest zones of range 2.9 ± 0.5 mm to 5.9 ± 0.5 mm was exhibited by methanol extract against all the strains. Close affinity was observed between the inhibitory zones of standard, oxytetracycline (6.4 ± 0.8 mm) and methanol extract (5.9 ± 0.5 mm) against *A.hydrophila.*

MIC determines the concentration of the drug (leaf extract) to which the pathogen is susceptible. The visible growth of *A.hydrophila* was prevented at 49 µg/ml concentration of methanol leaf extract of *A.ilicifolius*.

### Antioxidant activity

**Fig. 1:**
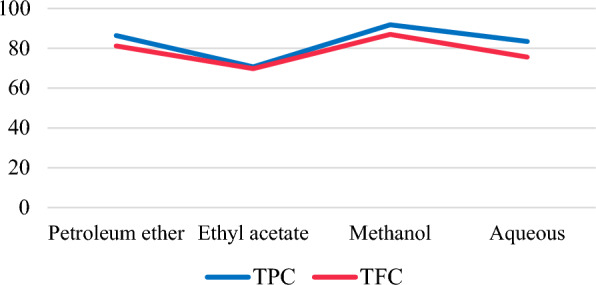
TPC and TFC of *A.ilicifolius* leaf extracts

**Fig. 2:**
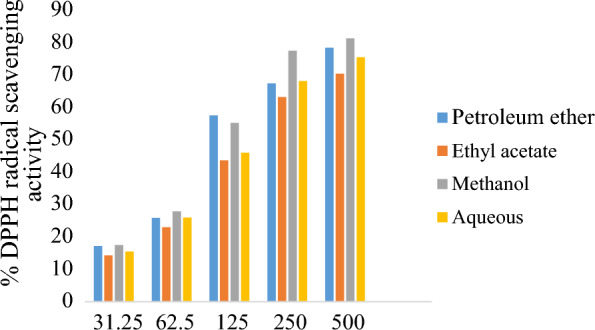
DPPH assay of *A.ilicifolius* leaf crude extracts, results were expressed in Ascorbic acid equivalents (µg/ml)

**Fig. 3:**
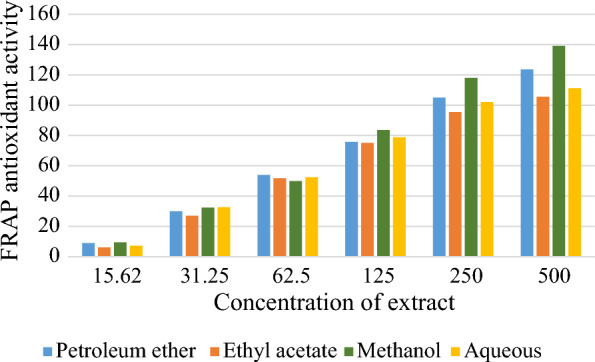
FRAP assay of *A.ilicifolius* leaf crude extracts, results were expressed in Ascorbic acid equivalents (µg/ml)

**Fig. 4:**
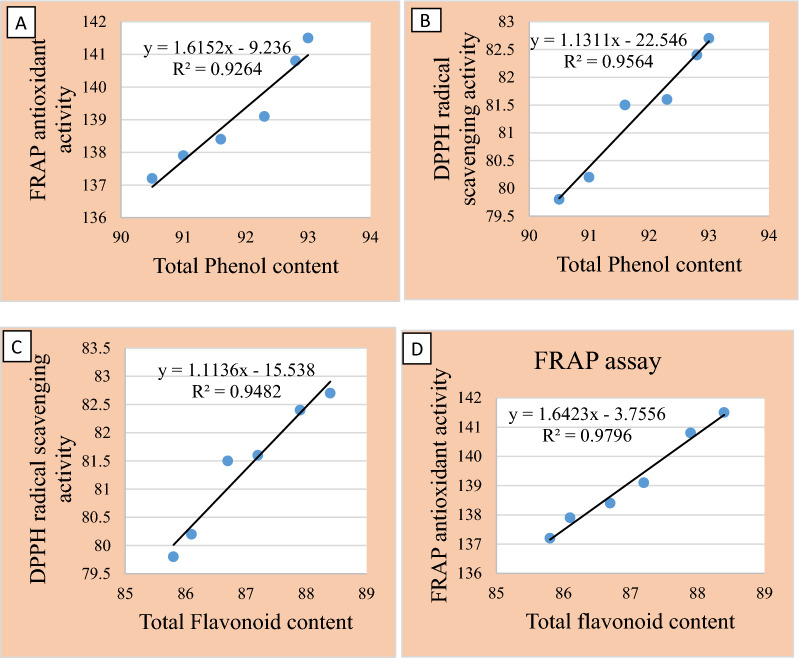
Correlation between TPC,TFC and antioxidant activity of methanol leaf extract **A** TPC vs FRAP, **B** TPC vs DPPH, **C** TFC vs DPPHand **D** TFC vs FRAP

Total phenol and flavonoid content were higher in methanol extract followed by petroleum ether, aqueous and ethyl acetate extracts (Fig. 1). The DPPH and FRAP antioxidant activity assays of the extracts was measured at different concentrations (Figs. 2 and 3). Among the extracts, methanol extract has resulted highest DPPH; 81.3 ± 1.0 AAE µg/ml and FRAP; 139.1 ± 1.5 AAE µg/ml activity at 500 µg/ml concentration. IC_50_ of the extracts ranged from 118.6 µg/ml by methanol to 164.8 µg/ml by aqueous extracts. Lower IC_50_ reflects higher antioxidant ability (Roy and Dutta 2021) inferring to the highest antioxidant activity of methanol. TPC and TFC of the all extracts has showed a linear correlation with the antioxidant activity (FRAP and DPPH) (Fig. 4 represents correlation of methanol extract).

## Separation and purification of the extracts

### Column chromatography

Phytocompounds in the methanol extract was separated. 14 fractions were obtained, grouped into five and pooled in accordance to their colour (Table 2). No bioactivity was observed in fraction B. Fraction E failed to inhibit the growth of *A.hydrophila* and D has no DPPH radical scavenging activity. Pooled fractions of A and C exhibited both antibacterial and antioxidant activity. Despite of the antibacterial and antioxidant activity shown by A and C, maximum inhibition of *A.hydrophila*, 7.5 ± 0.8 mm and %DPPH free radical scavenging activity, 83.2 ± 0.8% was reported in fraction A.

**Table 2:** Fractions, their antibacterial and antioxidant activity of *A.ilicifolius* Methanol leaf extract

Solvent system	Fractions	Code	Colour/ Characteristic	Zone of inhibition (mm) against *A. hydrophila*	% DPPH radical scavenging activity
100:0	1–3	A	White	7.5 ± 0.8	83.2 ± 0.8
90:10	–	–	–	-	–
80:20	4–6	B	Pale yellow	-	–
70:30	–	–	–	-	–
60:40	7–8	C	Yellow	2.1 ± 0.4	43.4 ± 1.1
50:50	–	–	–	–	–
40:60	–	–	–	–	–
30:70	–	–	–	–	–
20:80	9–11	D	Orange	1.2 ± 0.6	–
10:90	12–14	E	Yellow	–	38.7 ± 0.9
0:100	–	–	–	–	–

### Preparative TLC

Highest bioactivity of Fraction A might be due to the presence of potential antibacterial and antioxidant phytocompounds. For further separation of compounds pertained to bioactivity, this fraction was eluted in TLC, separated into three bands with different Rf values. Phytocompounds of each band was subjected to antibacterial and DPPH scavenging activity, (Table 3). AM1 and AM3 bands has lesser antibacterial and antioxidant activity. Band AM2 has highest antibacterial, 13.5 ± 1.2 mm and %DPPH radical scavenging, 88.1 ± 0.9% activity.

**Table 3:** TLC bands and their Antibacterial and antioxidant activity of *A. ilicifolius* Methanol leaf extract

Bands	Rf value	Zone of inhibition (mm) against *A. hydrophila*	% DPPH radical scavenging activity
AM1	0.17	1.6 ± 0.5	13.6 ± 1.3
AM2	0.24	13.5 ± 1.2	88.1 ± 0.9
AM3	0.29	2.4 ± 1.1	24.2 ± 1.8

### GC–MS

**Fig. 5:**
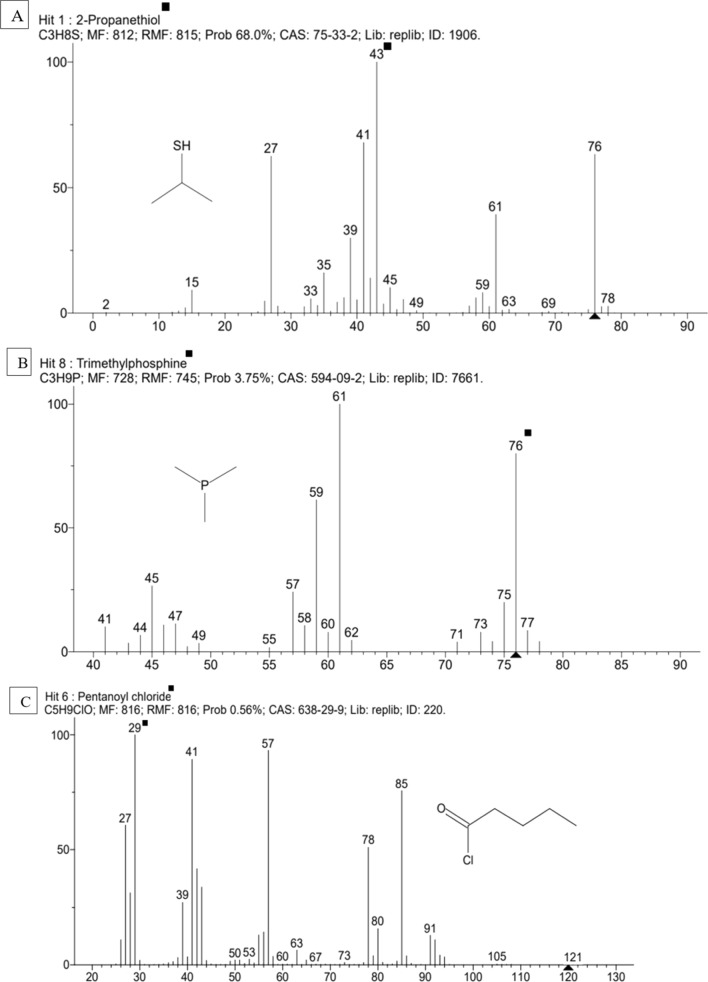

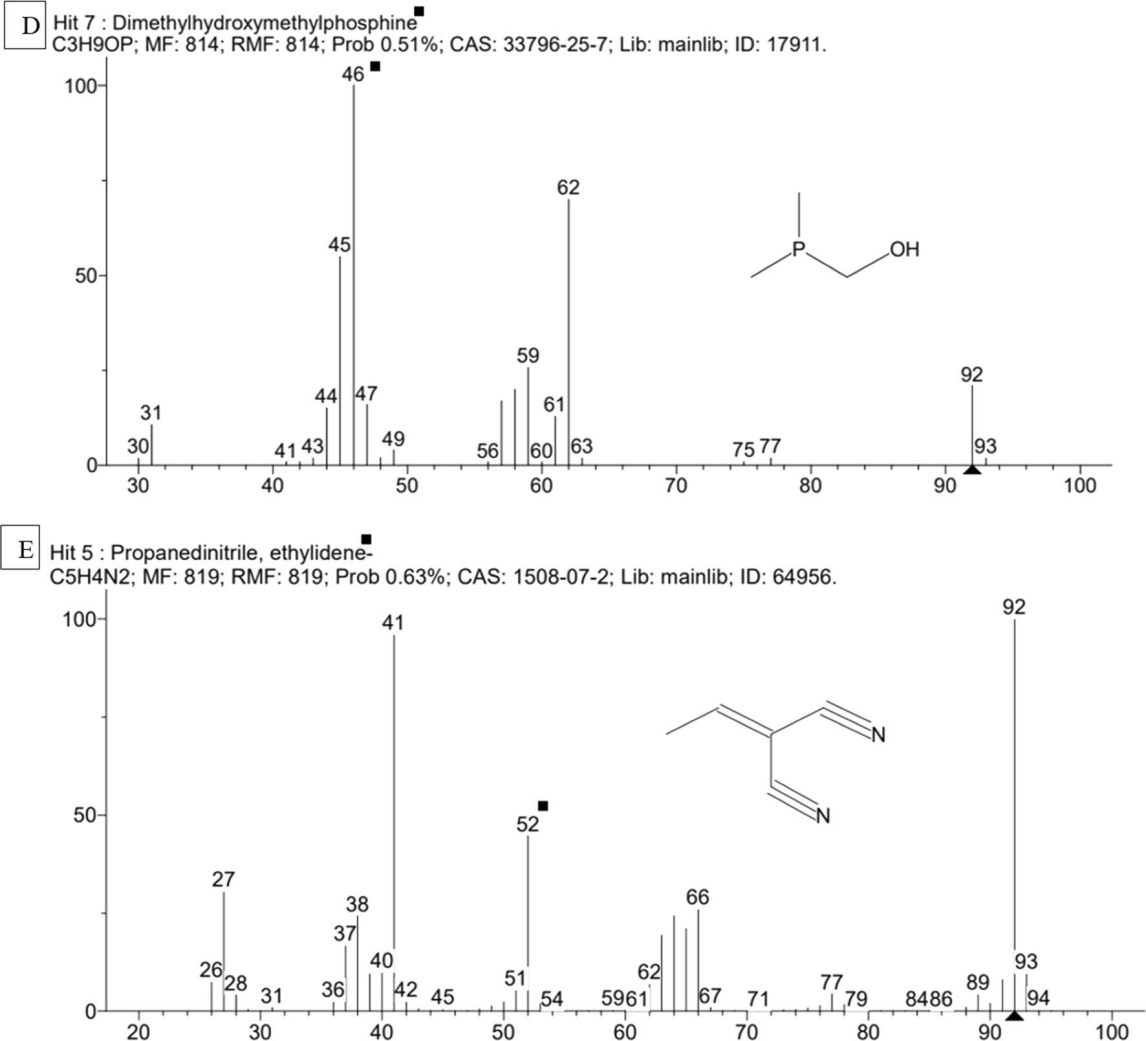
**A–E** GC–MS chromatogram of band AM2, *A.ilicifolius* methanol extract

GC–MS was adopted to characterise the compounds in AM2 responsible for antibacterial and antioxidant activity. Spectral finger print of the phytocompounds detected 5 compounds with corresponding retention time, molecular mass, molecular formula and peak area (Table 4 and Fig. 5A–E).

**Table 4:** GC–MS characterization of band AM2, *A.ilicifolius* methanol extract

S.no	Name of the compound	Synonym	Molecular Formula	Molecular mass	RT	Peak area %
1.	2-Propanethiol	Isopropanethiol, isopropylthiol, 2-Mercaptopropane, 2-propyl mercaptan	C_3_H_8_S	76.034	43.999	68.00
2.	Trimethylphosphine	Phosphine, trimethyl, trimethyl phosphorous	C_3_H_9_P	76.04	76.799	3.75
3.	Pentanoyl chloride	Valeryl chloride, valeroyl chloride, n-valeroyl chloride	C_5_H_9_C_lO_	120.03	29.999	0.56
4.	Dimethylhydroxymethylphosphine	(Dimethylphosphino)methanol	C_3_H_9_OP	92.04	46.999	0.51
5.	Propanedinitrile, ethylidene-	Hydroxymethyl-dimethylphosphine	C_5_H_4_N_2_	92.037	52.446	0.63

### In vivo studies

The therapeutic efficiency of *A.ilicifolius* crude and purified methanol leaf extract was assessed in in vivo against *A.hydrophila* infection in *L.rohita* fingerlings. Group I fingerlings, being control were healthy and exhibited 100% survival until end of the experiment. Fingerlings of group II to V, which were deliberately exposed to the *A.hydrophila,* showed behavioural and morphological alterations viz*.,* frequent hitting against the walls, change in swimming patterns, gill necrosis, septicaemia, haemorrhages and abrasion of fins, rotting of caudal region, abdominal dropsy, bulged eyes/ popped eye, red skin lesions and stick out of scales. These clinical manifestations were more pronounced in group II as the fingerlings were untreated with antibiotic or plant extracts and 0% survival was recorded. A clear external and internal signs were noticed in the post mortem of diseased fingerlings of all groups; accumulation of bloody fluids in intestine and externally body looks balloon, haemorrhages in hepatic tissue, kidney necrosis, hypertrophy and darkened spleen, disfigured internal organs. Oxytetracycline treated fingerlings showed 71% survival and those treated with crude and purified leaf extracts has showed 81% and 94% survival respectively (Additional file 1: Table S2).

**Fig. 6:**
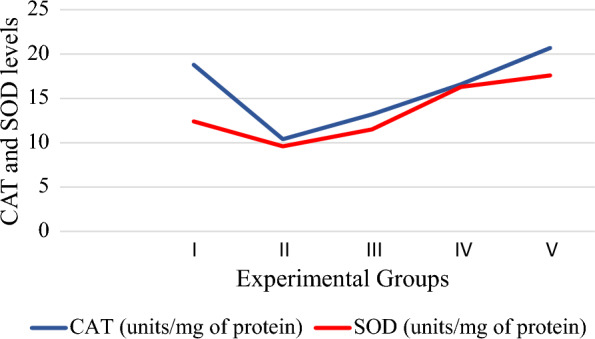
Levels of antioxidant enzymes CAT and SOD in experimental groups

The levels of hepatic enzymes CAT and SOD refers to capacity of free radical suppression. In experimental groups; fingerlings of group V recorded highest levels of CAT, 20.7 ± 1.2 and SOD, 17.6 ± 1.1. The enzyme levels in groups II to IV were lesser than the group V and group I. However, exception was noticed in SOD level of group IV (Fig. 6).

### Confirmation of *A. hydrophila*

A cluster of morphological and biochemical confirmatory tests confirmed the bacteria as *A.hydrophila* (Additional file 1: Table S3).

## Discussion

Frequent outburst of infectious diseases is one of the severe threats in aquaculture leading to monetary loss. For the last few decades, the therapeutic knowledge of treating these infectious diseases was confined to synthetic antibiotics and chemical drugs. Imprudent use of antibiotics inflict stress, dysbiosis of microbiota, metabolic malfunctioning, immune suppression, accumulation of residues in the tissues of fish (Limbu et al. 2021). On long run these therapeutic practices were increasing the pathogenic resistance, residual accumulation in the water, fish and consumers and there is a need to limit the usage of these drugs (Zhang et al. 2022). Medicinal flora and mangroves in specific are used in traditional practices of disease control and are therapeutic agents for ulcers, diabetes, tumors, skin diseases, inflammation etc. (Mitra et al. 2021), *A.ilicifolius* exhibit anticancer, antiviral, anti-inflammatory (Aisiah et al. 2022), *Excoecaria agallocha*; antioxidant, anti-filarial, antiviral (Abeysinghe 2010), *Bruguiera gymnorrhiza*; anti-analgesic, antioxidant, anti-diarrhoeal (Mahmud et al. 2017).

In the present study, *A.ilicifolius* leaf extracts functioned efficiently against the selected fish bacterial pathogens. Application of phytotherapeutics is bestowing positive results in the fish disease management (Zhu 2020). Leaf extracts of the selected mangrove plant has bioactive secondary metabolites considerably responsible for the anti-pathogenic activity. In similar with the present findings, tannins, saponins, carbohydrates, proteins, phenols, cardiac glycosides, were reported in the methanol (Ganesh and Vennila 2011; Pothiraj et al. 2021) and ethanol extract (Aisiah et al. 2022) of *A.ilicifolius*. Tannins, alkaloids and steroids inhibit the bacterial pathogenicity by inactivating bacterial adhesion cells, disrupting cell membrane composition and DNA synthesis, flavonoids and phenols scavenge the free radicals released during infection (Dewanto et al. 2019).

Phytochemicals in methanol leaf extract of the present study was highly antagonistic to free radicals and selected bacterial strains. Hydrophilic hydroxyl and amine groups of phenols, flavonoids and alkaloids aids in binding and penetration through the bacterial cell membrane (Sefa et al. 2020). The variation in the antibacterial activity among the extracts might be due to difference in qualitative phytochemicals or morphological variations of test pathogens (Giraldes et al. 2020). *A. marina* has showed the bactericidal activity against *P.aeruginosa, E.coli*, *S. aureus* (Okla et al. 2021), *R.apiculata* and *R.mucronata* against *A.hydrophila*, *V.harveyi*, *S.agalactiae* (Vittaya et al. 2022). MIC is the minimal concentration of the antipathogenic drug that arrest the visible pathogen growth (Kowalska-Krochmal and Dudek-Wicher 2021). Mangroves are potent inhibitors of bacterial pathogens, methanol leaf extract of *R.apiculata* showed significant MIC of 12.5 mg/ml and 6.25 mg/ml against *E.coli* and *S.agalactiae* (Laith 2021), MIC of methanol root extract of *B.gymnorrhiza* against *P.aeruginosa* and *S.enteric* was 48.27 µg/ml and 13.41 µg/ml (Acharya et al. 2020). Saad et al. (2011) reported the MIC of ethyl acetate, n-hexane, methanol leaf extracts of *L. littorea* against *S.aureus*, *B. cereus* and *E.coli* which ranged from 0.04 µg/ml to 1.11 µg/ml.

In the present observation, DPPH and FRAP assays depicted highest radical scavenging ability and the least IC_50_ in the methanol extract. Free radicals are usually produced in the body, during stress or diseased conditions and are scavenged by the antioxidants in the body (Beulah et al. 2022). (Sasidharan et al. 2011) conducted the studies on antioxidant potency of four mangrove plants, *R.mucronata, R. apiculata, A. officinalis* and *A. marina.* DPPH and FRAP results of the current study are in compliment with the *R.mucronata* leaf extracts (Beulah et al. 2022). Root and leaf extracts of *A.ilicifolius* act as potent antioxidants against free radicals (Firdaus et al. 2013).

Crude extracts are complex of phytocompounds from which impurities or unnecessary compounds must be dismissed to know the exact compound responsible for the antimicrobial activity (Sasidharan et al. 2011). Fraction A of column and band AM2 of TLC has elevated antibacterial and antioxidant activity than the crude methanol extract. This was in par with the studies of (Moovendhan et al. 2015) in *A.marina,* (Beulah et al. 2021) in *S.maritima* and (Divya et al. 2022) in *E.agallocha*. The compounds that are responsible for the antimicrobial activity are characterized and structurally elucidated by GC–MS (Konappa et al. 2020). 2-propanethiol is strong antioxidant and anti- bacterial agent (Demirkol et al. 2004), trimethylphosphine and dimethylhydroxymethylphosphine are the antibacterial agents (Wu et al. 2019), Pentanoyl chloride is an acylated compound that enhances the antimicrobial and antioxidant activity (Avitabile et al. 2018), propanedinitrile ethylidene act as antimicrobial agent (Puthran et al. 2019).

Biotic and abiotic stress favours the prevalence of *A.hydrophila* infection in fish. In the present study, resulted pathological symptoms in the *L.rohita* fingerlings of infected groups were in coherence with the *Aeromonas* infection in *C. carpio*, *C. catla* and *L.rohita* (Saharia et al. 2018). It is evident from the in vivo studies that the increased survival in groups IV and V reflecting the antibacterial efficiency of the some phyotocompounds. After first day of the treatment with the leaf extracts, groups IV and V has not showed any notable recovery changes, but on day 2 of the treatment, the feeding and swimming pattern were normalised and from day 3 with increase in the duration of the treatment the fingerlings slowly recovered from the gill necrosis, haemorrhages, septicaemia, bulged eyes, fin abrasion and abdominal dropsy. However, the observed mortality in the plant extract treated groups (Group IV and V) might be due to the stress imposed by the bacterial infection and more susceptibility of the fingerlings to the infection. Similarly, *R.mucronata* reported positive results in therapy against *Vibrio harveyi* in Nile tilapia (Mulyani et al. 2020). (Limbago et al. 2021) biologically proved the antibacterial potency of mangrove plants *Sonneratia alba*, *A. marina, A.officinalis,* and *B.cylindrica* against *Salmonella arizonae* in *Carassius auratus*.

CAT and SOD in the hepatic tissues act as first line of defence and controlling the levels of ROS (Abhijith et al 2016). In the current study, the antioxidant potency of phytocompounds was mirrored in the enhanced levels of CAT and SOD in the fingerlings of groups IV and V over group I, II and III, implying to the increased or decreased liver functioning that might be due to the variation in the free radical production (Rao 2006).

Phytotherapeutics is an efficient eco-friendly treatment for diseases without disturbing the environmental sustainability. Mangrove extracts can be an impressive natural approaches for the therapy of fish diseases in the place of antibiotics or chemical drugs as they can subsidise drug resistance and residual accumulation in fish and consumer. The present in vitro and in vivo studies proved and enhanced the therapeutic knowledge and application of phytocompounds as potent enough to act as antibacterial and antioxidant agents.

### Supplementary Information

**Additional file 1: Table S1.** Antibacterial activity of crude *A. ilicifolius *extracts. **Table S2.** Antibiotic, crude and purified extract treated fingerlings and % survival. **Table S3.** Morphological and Biochemical confirmatory tests for *A. hydrophila*.

## Data Availability

The data that support the findings of this study are available on request from the corresponding author. The data are not publicly available due to privacy ethical restrictions.

## Abbreviations

TPC: Total phenol content
TFC: Total flavonoid content
DPPH: α-Diphenyl-β-picrylhydrazyl
FRAP: Ferric reducing antioxidant power
CAT: Catalase
SOD: Superoxide dismutase
MHA: Mueller Hinton Agar media
MIC: Minimum inhibitory concentration
