# Supplementary material for: Anti-pathogenicity of Acanthus ilicifolius leaf extracts against A. hydrophila infection in Labeo rohita fingerlings
Source: AMB Express. 2023 Aug 20;13:86. doi: 10.1186/s13568-023-01595-y (PMC10440336; doi:10.1186/s13568-023-01595-y)
Supplement: Supplementary file 1 — Additional file 1: Table S1. Antibacterial activity of crude A. ilicifolius extracts. Table S2. Antibiotic, crude and purified extract treated fingerlings and % survival. Table S3. Morphological and Biochemical confirmatory tests for A. hydrophila. [file 13568_2023_1595_MOESM1_ESM.docx]

**Additional file 1: Table S1**

| Bacteria | Zone of inhibition (mm) | | | | |
| --- | --- | --- | --- | --- | --- |
|  | Oxytetracycline | Petroleum ether | Ethyl acetate | Methanol | Aqueous |
| *P.aeruginosa* | 5.1±0.9 | 3.6±1.2 | 3.2±0.8 | 4±0.3 | 3.4±0.6 |
| *A. hydrophila* | 6.4±0.8 | 4.8±0.6 | 3.4±0.4 | 5.9±0.5 | 3.8±1.0 |
| *S. aureus* | 4.1±1.2 | 2.7±1.0 | 2.1±0.4 | 3.5±0.7 | 2.3±0.9 |
| *B. subtilis* | 3.8±0.6 | 2.2±0.6 | 1.4±0.7 | 2.9±0.5 | 1.9±0.8 |

**Additional file 1: Table S2**

| Group | Treatment | Survival percentage |
| --- | --- | --- |
| Group I | Control  Non treated | 100% |
| Group II | Negative control  Non treated | 0% |
| Group III | Oxyteyracycline | 71% |
| Group IV | Crude extract | 81% |
| Group V | Purified | 94 % |

**Additional file 1: Table S3**

| Test | Result/ observed colour |
| --- | --- |
| Morphological | |
| Shape | Rod shaped |
| Gram’s staining test | Red colour |
| Biochemical | |
| Oxidase test | No change in the colour. |
| Catalase test | No bubbling formation. |
| Indole test | No change in the colour. |
| Methyl red test | Yellow colour |
| Citrate utilization test | No change in the colour |
| Gelatin liquefaction test | Solidification |
| Motility test | No hazy growth and growth was confined to stab line. |
| Voges Prokauer test | No change in the colour. |

**Legends for tables**

**Additional file 1: Table S1.** Antihttps://doi.org/10.1186/s12933-023-01803-wbacterial activity of crude *A. ilicifolius* extracts

**Additional file 1: Table S2.** Antibiotic, crude and purified extract treated fingerlings and % survival

**Additional file 1: Table S3.** Morphological and Biochemical confirmatory tests for *A. hydrophila*
